# Comparison of Blastema Formation after Injury in Two Cephalopod Species

**DOI:** 10.17912/micropub.biology.000946

**Published:** 2023-09-19

**Authors:** Carlos Chavez Ramirez, Miya Khoo, Marco Lopez G, Sophie Ferguson, Sarah Walker, Karen Echeverri

**Affiliations:** 1 University of Chicago, Chicago, Illinois, United States; 2 Eugene Bell Center for Regenerative Biology and Tissue Engineering, Marine Biological Laboratory, Woods Hole, Massachusetts, United States

## Abstract

Regeneration is the ability to functionally replace significant amounts of lost tissue or whole appendages like arms, limbs or tentacles. The amount of tissue that can be regenerated varies among species, but regeneration is found in both invertebrate and vertebrate animals. Cephalopods have been broadly reported in the literature to regenerate their arms. There are over 800 species of Cephalopod; however, regeneration has only been documented in the literature in a few species (1). Here we compare arm regeneration in two species of cephalopod, the *Octopus bimaculoides* and the hummingbird bobtail squid *Euprymna berryi.*

**Figure 1. Comparison of the early stages of regeneration in two species of Cephalopod:**
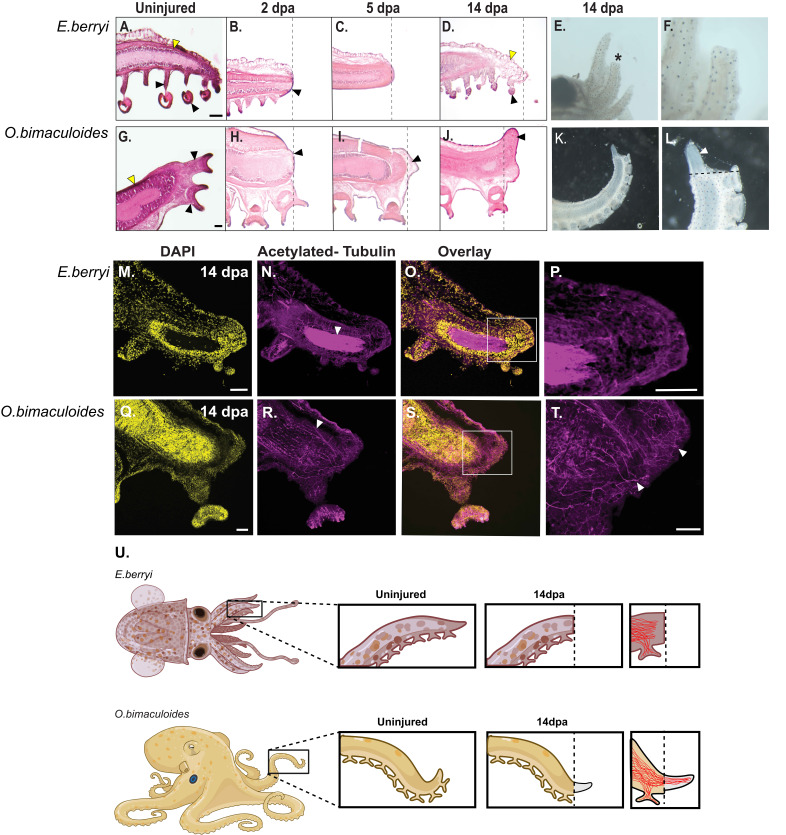
**(A-D)** Haematoxylin and Eosin (H&E) staining’s of sections of the unamputated and injured arm of *Euprymna berryi* . By 14 days post amputation (dpa) loose mesenchymal like tissue (D; yellow arrow) is observed at the tip of the injured arm. **(E,F)** Whole animal images of the injured arm at 14dpa. Asterisk in E indicates the amputated arm which as a different morphology to the adjacent uninjured arm, (F) is a higher magnification image of the amputated arm in (E). **(G-J)** H&E staining of the uninjured and injured arm of the *Octopus bimaculoides.* By 5dpa a clear mound of cells is visible adjacent to the mature differentiated tissue (I; black arrow), this mound of cells has expanded by 14dpa (J; black arrow). **(K,L)** Whole animal images of the amputated limb at 14dpa. A blastema-like structure with a different morphological appearance to the mature arm tissue and lacking chromatophores is visible at the end of the amputated arm (K), higher magnification view (L; white arrow). Scale Bar = 100µm. Dotted lines indicate the plane of amputation. **(M-T)** Anti-acetylated tubulin staining of axons in the amputated arm at 14dpa. White arrows (N, R) indicate the mass of dense nerve bundles seen in the tentacles of both species. White arrows (T) indicate nerves extending the length of the regenerated tissue in the Octopus at a higher magnification. Scale bar = 100µm. **(U)** Summary of *E.Berryi* and *O.bimaculoides* responses to tentacle amputation. By 14dpa, no regenerative outgrowth is shown in *E.Berryi,* while a regeneration blastema has formed in *O.bimaculoides* . The nerves in the amputated tentacle of the *E.Berryi* fail to extend the entire length of the tentacle, while the nerves extend into the newly regenerated tissue of the *O.bimaculoides* tentacle.

## Description

Cephalopods are known to be capable of arm regeneration; several descriptions exist of this phenomenon, however, these are mainly limited to various adult octopus species (1). We have now examined arm regeneration in the juvenile *Octopus bimaculoides* and in the juvenile hummingbird bobtail squid, *Euprymna berryi* . We have used haemotoxylin and eosin staining on cryosections of the fixed arm tissue at 2, 7 and 14 days post-amputation (dpa) to examine the morphology of the tissue post-injury. The unamputated arms in both species consist of a slender, elongated structure that gradually tapers off (A, G; yellow arrows), with suckers evenly spaced along the arm right up to the tip (A, G; black arrows). The arms were amputated after the last two suckers and then the regenerating tissue was fixed at 3, 5 and 14 dpa. At 2 dpa in both species the wound closed, and the presence of thickened tissue was observed by the deeper purple stain covering the cut surface (B, H; black arrows). By 5 dpa in the octopus, we observed a mound of cells accumulating at the cut site (I; black arrow), however, this was not seen in the bobtail squid (C). At 14 dpa we see further growth of a mound of cells adjacent to the amputation site in the octopus (J; black arrow); this structure of cells with a very similar morphology is reminiscent of a blastema, which describes a mound of proliferating undifferentiated cells or post-mitotic progenitors, observed adjacent to the injury site in species known to be capable of regeneration like salamanders, zebrafish and planaria (2-4). The blastema like structure observed here is very similar to what has been documented in adult arm regeneration in another octopus species, *O.vulgaris* (5-7). Interestingly, in the bobtail squid at 14 dpa we observed fragile mesenchymal-like tissue (D; yellow arrow) and that a sucker is now present close to the end of the amputated arm (D; black arrow). The morphology observed in the bobtail squid at 14 dpa suggests that the tissue has merely rearranged itself rather than a process of epimorphic regeneration. Images of the intact arms by brightfield microscopy suggest little outgrowth of tissue at 14 dpa in the squid (E, F, asterisk in E indicates the amputated arm).

In the octopus at 14 dpa, the blastema-like structure continues to grow (J; black arrow). Wholemount images show a clear difference in morphology of the tissue growing at the end of the amputated arm versus the mature tissue (K, L; white arrow indicates newly regenerated tissue).

We also carried out antibody staining using an acetylated tubulin antibody to document nerve regeneration at 14 dpa. In both species, we observed dense networks of nerves (N, R, white arrows). In the bobtail squid, we see that the nerves are present but very few fibers extend right to the tip of the amputated arm (N, P); however, in the octopus, we observe that the nerves extend into the area of regenerating tissue (R, T; white arrows).

The nerve dependency of regeneration, whereby the nerves must reach into the area of the regenerating tissue and potentially provide essential growth factors such as neuregulin (NRG1), has been documented in several species, including salamanders and newts for arm and heart regeneration (8-18). The lack of robust nerves extending into the growth zone in the *E. berryi* may suggest a lack of regeneration in these animals. However, it is also possible that regeneration takes much longer in these animals. In the future it would be interesting to observe these animals for longer to determine if *E.berryi* eventually will in fact regenerate but the process simply takes much longer. We have also amputated the limbs of another Cephalopod species, the pyjama squid, *Sepioloidea lineolate* and observed a phenotype similar to *E.berryi* where no blastema is formed during this timeframe. Due to the difficulties in obtaining and housing cephalopods most regeneration studies to date have been carried out on adult *O.vulgaris* and there is more limited information of regeneration in other related cephalopods. All of these animals we have studied in these experiments are at a juvenile stage but all have different body sizes, different life spans and reproductive cycles, however it is unknown how these parameters may relate to their regenerative abilities.

As technology has advanced and the life cycle has been closed in laboratories for different cephalopod species (19) they become more accessible for studying at different points in their life cycle. The sequencing of the genomes of different cephalopod species (20-25) and the development of the first genetically modified Cephalopod (26), where gene knock-out is possible will now enable the testing of gene function during arm regeneration in these species. The recent establishment of an albino squid line will enable cell tracking in vivo during arm regeneration (27).

Overall our results suggest that a robust blastema is formed in the *Octopus bimaculoides* in response to arm amputation; however, the *Euprymna berryi* appears to instead heal over the injured tissue, but we did not observe the classical blastema-like structure at 14dpa suggestive of a pro-regenerative response (U).

## Methods

Animals

All animals used in this study were bred by the Marine Biological Laboratory (MBL) Cephalopod program, usage of animals for regeneration experiments was carried out with oversight provided by the Institutional Animal Care and Use Committee . Of the MBL. Two species were used, *Octopus bimaculoides* and *Euprymna berryi* . All animals were post-hatchling, 4-6 weeks old. Animals were fed daily with shrimp, and the water was changed post feeding. Animals were housed in individual containers and maintained in local seawater for the duration of the studies.

Before amputations, cephalopods were anesthetized. *O. bimaculoides* were placed in 2% ethanol, while *E. berryi* were placed in 7.5% MgCl _2_ . After confirming that the animals were relaxed by reduced movement and response to touch, a portion of an arm corresponding to approximately two suckers’ length was amputated. One arm on each side of the body of each animal was amputated.

Tissue Fixation

At 2, 5, 7, and 14-dpa, four animals of each species were fixed. The *O. bimaculoides* specimens were treated with 100% MgCl _2_ for euthanization, being placed in 4% paraformaldehyde (PFA) for one hour after euthanization. *E. berryi* were euthanized using 100% ethanol. Harvested tissue was immediately placed into 4% PFA for fixation.

Cryosectioning

Tissue samples were washed in phosphate buffered saline (PBS) after fixation and then placed in 30% sucrose overnight at 4°C. The next day, samples were embedded in TissueTek O.C.T Compound for sectioning. Twenty-micron sections were collected using a Leica CM1850 cryostat.

Histological Staining

The samples were rehydrated in PBS, then washed 2 x 5mins in tap water. Slides were then incubated in haematoxylin stain for 6 mins. Next, sections were washed in tap water for 3 mins followed by a quick wash in a mild acid solution, followed by a water and ethanol wash. The sections were then incubated in eosin for 5 mins and washed in 95% and 100% ethanol. Sections were finally dipped in Xylene and mounted using Richard-Allan Scientific Mounting Medium for imaging.

Antibody staining

Sections were permeabilized using PBS plus 0.5% Triton X-100 for 30mins at room temperature. Blocking was caried out at room temperature for 1 hour using PBS plus 10% goat serum. The primary antibody (anti-acetylated tubulin Sigma T6793) was diluted 1:500 in blocking buffer (PBS plus 10% goat serum) and incubated overnight at 4°C. Sections were then washed 3 x 10 mins in PBS plus 0.1% Tween-20 (PBST). Sections were incubated in secondary antibody (anti-mouse Alexa 568) for 1 hour, washed in PBST and then incubated in DAPI for 15 mins. Lastly, sections were washed in PBST, covered in mounting media (Sigma F4680) and cover slipped.

Imaging

Images of tentacle regeneration in live animals and fixed sections used in histological staining were imaged using a Zeiss Discovery V8 Stereo microscope. Fluorescent microscopy images were taken on a Zeiss LSM780 using a 10x objective lens.

## Reagents

**Table:** 

**Reagent**	**Catalog Number**
Acetylated Tubulin antibody	Millipore Sigma, T6793
Alexa Fluor 568 goat anti-mouse secondary antibody	Invitrogen, A11011
